# Notices, Comments, &c.

**Published:** 1881-05

**Authors:** 


### NOTICES, COMMENTS, ETC.

The American Pædological Society will meet in New York City, June 13th, the day before the meeting of the Institute. Further particulars will be given later. A grand time is anticipated, and we hope to show the world that homœopaths are *the* physicians for children.

J. C. Duncan, President, Wm. Owens, Vice President,
E. Cranch, Secretary.

---

Sidney, O., March 15.

Dear Doctor,—You are cordially invited to attend the Seventeenth Annual Session of the Homœopathic Medical Society of Ohio, to be held in Toledo, May 10–11. The coming session promises to be one of the most interesting and profitable ever held by the Society, as we have the promise of a large number of papers from noted physicians of the State and the prospect of a large attendance, not only from our own, but from neighboring states. Your presence is earnestly desired. Respectfully,

H. E. Beebe, M. D., Secretary.

---

The International Hahnemannian Association meets at same place and time, as the Institute, "And don't you forget it."

---

### HAS NOT HEARD OF JAEGER.

High Potencies.—At the recent meeting of the New York State Homœopathic Society, Dr. H. M. Paine had the candor and boldness to say: "Our experience in the use of high potencies is based, as Hahnemann's was, on theoretical grounds only. It is one of the most singular forms of idealism ever seriously entertained by the medical profession. I firmly believe that when our reputed cures are reported in connection with all cases treated we shall find that their frequency is not greater than those of daily occurence, without medicine of any kind."—*Medical Reeord.*

---

### AGGRAVATION FROM THE 200TH.

We recently sent a gentleman some powders suitable to his case. The first *two powders* contained *Nux vom.* 200th., the rest Sac. Lac. In reply to our letter and medicine, he wrote: "Began taking your powders last evening and took three before 10 p.m. I find they affect my back, my weak point, as when I had taken a dose of *Strychnine,* though not to so great a degree. I thought I had best suspend taking more until I heard from you." This gentleman is an allopath. He knew nothing as to the contents of the powders.—Ed.

